# Right and Left Colorectal Cancer: Differences in Post-Surgical-Care Outcomes and Survival in Elderly Patients

**DOI:** 10.3390/cancers13112647

**Published:** 2021-05-28

**Authors:** Irene Mirón Fernández, Santiago Mera Velasco, Jesús Damián Turiño Luque, Iván González Poveda, Manuel Ruiz López, Julio Santoyo Santoyo

**Affiliations:** 1 Department of General, Digestive and Transplant Surgery, Malaga Regional University Hospital, Malaga’s University, 29010 Málaga, Spain; jesusd.turino.sspa@juntadeandalucia.es (J.D.T.L.); julio.santoyo.sspa@juntadeandalucia.es (J.S.S.); 2 Colorectal Unit, Department of General, Digestive and Transplant Surgery, Malaga Regional University Hospital, 29010 Málaga, Spain; santiago.mera.sspa@juntadeandalucia.es (S.M.V.); ivan.gonzalez.sspa@juntadeandalucia.es (I.G.P.); manuel.ruiz.lopez.sspa@juntadeandalucia.es (M.R.L.)

**Keywords:** cancer, colon, survival, differences

## Abstract

**Simple Summary:**

The objective of this investigation is to analyze the differences between right and left colon cancer survival and test if these differences have transcendental importance for assistance to improve the survival and quality care of these patients. The results show that both entities are significantly different in terms of evolution, progression, complications and survival. Patients with right colon cancer have a worse prognosis, even in the early stages of the disease, due to more advanced N stages, a larger tumor size, more frequently poorly differentiated tumors and a greater positivity of lymphovascular invasion than left colon cancer. Improvement of the prognosis can be implemented mainly by reducing the specific mortality of colon cancer by achieving early detection and also stratified and personalized by location and age of onset, as well as surgical and oncological treatment of these patients.

**Abstract:**

(1) There is evidence of the embryological, anatomical, histological, genetic and immunological differences between right colon cancer (RCC) and left colon cancer (LCC). This research has the general objective of studying the differences in outcome between RCC and LCC. (2) A longitudinal analytical study with prospective follow-up of the case–control type was conducted from 1 January 2010 to 31 December 2017 including 398 patients with 1:1 matching, depending on the location of the tumor. Inclusion criteria: programmed colectomies, 15 cm above the anal margin, adults and R0 surgery. (3) Precisely 6.8% of the exitus occurred in the first 6 months of the intervention. At 6 months, patients with LCC presented a mean survival of 7 months higher than RCC (*p* = 0.028). In the first stages, it can be observed that most of the exitus are for patients with RCC (stage I *p* = 0.021, stage II *p* = 0.014). In the last stages, the distribution of the deaths does not show differences between locations (stage III *p* = 0.683, stage IV *p* = 0.898). (4) The results show that RCC and LCC are significantly different in terms of evolution, progression, complications and survival. Patients with RCC have a worse prognosis, even in the early stages of the disease, due to more advanced N stages, larger tumor size, more frequently poorly differentiated tumors and a greater positivity of lymphovascular invasion than LCC.

## 1. Introduction

Colorectal cancer (CRC) is currently one of the most frequent neoplasms in the world and with few differences between men and women [1]. This tumor is more prevalent in older people. The average age of presentation is 70–71 years, and a great percentage of patients are over 50 years old at the time of diagnosis, although it can also appear in younger patients. In relation to sex, it affects men and women almost equally [2]. According to the Globocan estimation for 2020, the most frequently diagnosed cancers in Spain in 2020 will be those of the colon and rectum (44,231 new cases), prostate (35,126), breast (32,953), lung (29,638) and urinary bladder (22,350). Moreover, the Spanish Agency against Cancer reports that colorectal cancer is the most prevalent cancer in both sexes, with a percentage of 10.7%, and is the second tumor with the highest mortality in Spain [3].

Colorectal cancer is in Spain the most diagnosed neoplasia (15% of total tumors) and the second cause of death due to cancer in the country [4]. More than 37,000 persons are diagnosed with this cancer every year in the country, and it is the first in the battlefield if summing up both sexes. It is also the second cause of death, with an estimated rate of 15,000 deaths per year [5]. Worldwide, CRC is the third malignant tumor in frequency and the second cause of death due to cancer. Its incidence is a global health problem, taking third place worldwide and the second in mortality registered by Globocan [6,7].

Having established its importance due to its incidence and prevalence, it is also known from current evidence that there are embryological, histological, genetic and immunological differences between right and left colon cancer [8,9,10].

Most of the tumors are sporadic, and part of them share known modifiable risk factors belonging to human lifestyle: obesity, sedentary behavior, poor or bad diets, alcohol intake, tobacco habits and economic situation [11,12].

Moreover, current studies have described differences in epidemiology, clinical features and biology between left (LCC) and right colon cancer (RCC) that generally affect old people [13].

Characteristically, right-sided colon cancer is associated with iron deficiency anemia, advanced stage, advanced age and female sex, presenting with exophytic lesions that grow inward. Left-sided colon cancer, however, tends to be infiltrative and stenotic, leading to obstruction and presenting urgently [14].

Embryologically, the right colon (cecum, ascending and the proximal 2/3 of the transverse colon) originates from the midgut, while the left colon (distal 1/3 of the transverse colon, descending, sigmoid and rectum) originates from the hindgut. This difference is reflected by the distinct blood supply of the right and left colon, suggesting variants in the physiopathology of cancer and type of surgery to be performed. As such, RCC and LCC could represent two different forms of disease [14].

Historically, many studies have reported different oncological results according to tumor location, with worse outcomes in right-sided colon cancer [15]; however, it is not yet clear because some studies show better prognosis in these patients [16].

The general objective of this surgical and assistance investigation is to analyze the differences between RCC and LCC survival and to test if these differences have transcendental importance for assistance to improve the survival and quality care of these patients.

## 2. Materials and Methods

A prospective, longitudinal study was performed including patients diagnosed with colon cancer from 1 January 2010 through 31 December 2017. There were two different groups: one group with patients who have right colon cancer and another group with patients who have left colon cancer, with 1:1 matching. There were two periods of follow-up at six and twelve months in the postoperative course. The minimal follow-up was twelve months for every patient [17].

The study took place in a third-level regional hospital (more than 1000 beds and all the specialties) in Málaga, which belongs to the Spanish National Health System.

Right colon cancers (RCCs) were identified as tumors in the cecum, ascending colon, hepatic flexure and transverse colon. Left colon cancers (LCCs) were identified as tumors of the descending colon and sigmoid colon. Rectal cancers were excluded. Surgery of left colon cancer was performed with intracorporeal anastomosis; for right colon cancer, anastomosis was extracorporeal.

Inclusion criteria included: elective resection of colon cancer, cancers located 15 cm above the anal margin, patients over 18 years of age, autonomy to sign the informed consent for the procedure and R0 resection (macroscopic and microscopic margins free of disease) at the first surgery. Exclusion criteria included: patients without the capacity to sign the informed consent, emergency surgery, palliative surgery, presence of carcinomatosis, synchronous or metachronous lesions, recurrent tumors and initial surgeries with R1/R2 resections. All of the patients were treated with the Enhanced Recovery After Surgery (ERAS) protocol with respect to presurgical diets, antibiotic prophylaxis (amoxicillin–clavulanic acid 2 g intravenous), thromboembolic prophylaxis with low-molecular-weight heparin postoperatively on Day 1 and postoperative diet), according to a previous procedure [18].

Based on these inclusion criteria, the sample consisted of a total of 449 patients who were divided into two groups, and we performed 1:1 stratified matching between Group 1 patients with RCC (*n* = 199) and Group 2 patients with LCC (*n* = 199). All patients underwent curative surgery for right-sided and left-sided colon cancer and were identified from the Surgical Service Database.

Variables assessed in our study included: age, sex, body mass index (BMI), comorbidities, history of prior surgery, histopathology, tumor stage according to the TNM classification of colorectal carcinoma (AJCC 8th edition), Portsmouth POSSUM score of morbidity and mortality (P-POSSUM) [19], postoperative complications, laparoscopic versus open surgical technique, operative time, ratio of positive lymph nodes to total lymph nodes harvested, length of stay, survival at six and twelve months and cause of death. As quality markers, length of stay and mortality rate were used [20].

### Statistical Analysis

For analysis and processing of the data, SPSS version 22.0 was used. The variant analysis was performed using the X² test and Fisher’s exact test. Statistical significance was established as a value of *p* < 0.05. Categorical variables were analyzed using the X² test or Fisher’s exact test, and continuous variables were analyzed using the Student’s *t*-test or Mann–Whitney U-rank test. For survival analysis, the Kaplan–Meier method was used in both the six- and twelve-month control. This was carried out by applying the actuarial and Kaplan–Meier methods to estimate the probability of survival and the risk of death; the log-rank test and the Wilcoxon statistic were used to evaluate the statistical differences between the observed survival curves of the categories of each categorical variable, and the Cox regression model was used to identify the prognostic factors of the risk of death, as it is done by all the Spanish Cancer Records [21].

## 3. Results

### 3.1. Epidemiologic Characteristics of the Patients

The difference between the epidemiologic variables in the two groups is reflected in Table 1. The mean age was 67–70 years. We observed statistically significant differences in age (*p* < 0.001), mortality (P-POSSUM; *p* = 0.027) and almost significant differences in the history of previous surgeries (*p* = 0.05). All of this is in favor of RCC, which is more likely in elderly people.

When stratifying the sample according to the TNM system in Table 2, it is observed that 39.7% of patients (158) are in stage II, followed by 26.1% (104) in stage III, 25.4% (101) in stage I and finally 35 patients (8.8%) in stage IV of the disease. To facilitate the analysis of survival, patients were grouped according to the stage of their neoplasm in the local stage (I–IIa), where we found 244 patients (61.3%) and advanced stage (IIb to IV), with 154 patients (38.7%).

The number of isolated nodes was higher in the RCC group than in the LCC group, these differences being statistically significant (*p* < 0.001). However, nodal involvement by the tumor did not show statistically significant differences between the two cancers (*p* = 0.857).

### 3.2. Postoperative Course

The surgical approach was conventional in 55.3% (*n* = 110) in the RCC group and 21.6% in the LCC group (*n* = 43), and the laparoscopic approach was mostly used in 70.3% in the LCC group (*n* = 140) against 40.7% (*n* = 81) in the RCC group, these differences being statistically significant (*p* < 0.001). In the surgical procedure carried out, LCC surgery was more frequently performed by laparoscopy without conversion (*p* = 0.022; OR 0.259), being more likely to find advanced tumors intraoperatively in this location (*p* = 0.004; OR 0.267) with, therefore, increased surgical time (*p* = 0.036; OR 1.006).

Table 3 shows postoperative complications according to both study groups. The majority of complications in the RCC group was 50.8% (*n* = 101) compared to the 26.1% in the LCC group (*n* = 52) with a value of *p* < 0.001. This is reflected by a higher rate of superficial surgical wound infection, the presence of intraabdominal abscess and postoperative paralytic ileus. These complications led to a longer postoperative stay of the group of RCC patients of 13.6 days (SD ± 11.9) compared to 9.4 days (SD ± 8.2) in the LCC group, with statistically significant differences (*p* = 0.004).

In relation to the variables studied that show significant statistical association, multivariate analysis establishes that the older the patient, the greater the probability of suffering from cancer of the right side (*p* = 0.005; OR 0.965).

In the surgical procedure carried out, LCC surgery was more frequently performed laparoscopically without conversion (*p* = 0.022; OR 0.259), and it was more likely to find advanced tumors intraoperatively in this location (*p* = 0.004; OR 0.267) with, therefore, an increase in surgical time (*p* = 0.036; OR 1.006).

However, in relation to the complications, it is observed that there is a greater probability that they occur when the patient suffers from RCC (*p* = 0.005; OR 2.073), with an associated increase in the probability of developing a postoperative intra-abdominal abscess (*p* = 0.041, OR 4.388).

### 3.3. Survival Analysis

**Figure 1:**
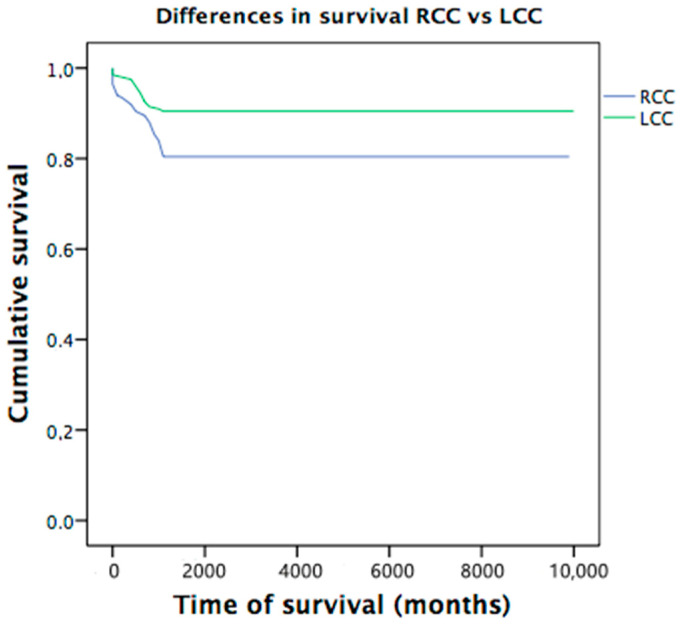
Differences in survival between RCC and LCC.

A total of 85 deaths were observed, 27 of which (6.8%) occurred in the first 6 months and 58 cases (14.6%) 12 months after the intervention. At 6 months, average overall survival of 93% (IC 95%: 90.9–95.7) was observed, and at 12 months, it was reduced to 86.2% (IC 95%: 82.9–89.5). According to the location, survival times are shorter in RCC at 6 and 12 months, with statistically significant differences (*p* = 0.005) (Figure 1). Statistically significant differences are also observed between locations according to local stage group (*p* = 0.007) and stage I patients (*p* = 0.022) with higher mortality in the RCC group. There were no significant differences in the cause of exitus (cancer-related or non-cancer-related) between the two groups (Table 4).

When analyzing each stage of the disease, it is observed that at 6 months, there are significant differences in mortality for stages I (*p* = 0.021) and II (*p* = 0.014), which are higher in the RCC group; however, there are no significant differences in stages III (*p* = 0.683) and IV (*p* = 0.898). In the analysis at 12 months, these differences are maintained, with higher mortality in RCC for stages I (*p* = 0.022) and II (*p* = 0.062), and there are differences between stages III (*p* = 0.616) and IV (*p* = 0.124).

The study of mortality at 6 months found, in the group of patients with neoplastic exitus, similar mean survival times in both locations (RCC 61.2 ± 2.7; LCC 62.2 ± 3.3) (*p* = 0.647). In the group with another cause of death different from the neoplasia, there were differences in the average survival time of both locations without these being significant (RCC 39.1 ± 6.9, LCC 74.05 ± 9.9) (*p* = 0.110).

At 12 months in the patients who died from cancer, similar average survival times were found in both locations (RCC 46.8 ± 5.4; LCC 55.3 ± 4.7) *p*= 0.422. However, in the group that died of a non-neoplastic cause, there were differences in the mean survival times according to the location in favor of the left location (RCC 32.23 ± 6.69; LCC 69.105 ± 10.44) very close to significance (*p* = 0.066).

Postsurgical hospital stay was 9.36 in the LCC group and 13.61 in the RCC group, which means that the LCC group meets this quality indicator of the basic and specific quality standards of the Accredited Units of Colorectal Cancer Surgery [20].

## 4. Discussion

When analyzing the epidemiological variables of the patients in the study, the existence of statistically significant differences in relation to age between the two groups is verified (*p* < 0.001). This observation coincides with the findings presented by other authors [13,14,15]. In fact, one study showed a positive linear correlation between the age of the patient and the proximity of the tumor to the ileocecal valve [15].

Stratifying CRC according to the criteria of location and age of onset is important and transcendental not only because it correlates with the resulting molecular-based categories, which were described in the 1990s [8] but also because, with confirmation in larger studies, new therapeutic algorithms can be defined according to their subclassification [22].

A recent review emphasized the differences between tumors in the proximal (right side) and distal (left side) colon because they present different molecular and histological characteristics that determine the treatments to be used and, therefore, the effectiveness of chemotherapy treatments [10].

In relation to sex, as with the results presented by other authors, no differences between the two groups are objectified [14]. However, this contrasts with the results presented by others [15], which do show a significantly higher incidence of RCC in women (*p* = 0.01 and *p* = 0.023, respectively) with late presentation subtypes [23].

With regard to BMI values, no differences were observed between the two groups in the study (*p* = 0.997), as was the case in a previous study [14]. Analysis of the existence of comorbidities in the patients in this study shows no significant differences between the two cancers (*p* = 0.08), an aspect observed in a study in which the group of patients with RCC presented more previous diseases in a statistically significant manner (*p* < 0.001), an observation that could be due to the different sample sizes between the studies [15].

In relation to the number of isolated nodes grouped according to the quality standards of the Washington MK [24], there are statistically significant differences between both groups of patients (*p* = 0.001); however, neither the degree of affectation of these nodes by the tumor nor its tumoral study present statistically significant differences (*p* = 0.857 and *p* = 0.71, respectively). This could be explained by the constant change in the person who performs the histopathological analysis.

When assessing the overall surgical complications in both groups, we found a higher rate in patients who had undergone significant RCC surgery (*p* < 0.001) and specifically in surgical wound infection (*p* < 0.001), as well as intra-abdominal abscess (*p* = 0.02). These differences could be explained by the type of anastomosis, which was intracorporeal in the left colon cancer but extracorporeal in the right side (extracorporeal anastomosis has more risk of wound infection). These results are not consistent with the results provided by other authors [25].

Unlike the results presented in a previous study [15], this study found a significantly higher incidence of postoperative ileus in patients with right-sided pathology (*p* < 0.001). This fact could be related to the lesser laparoscopic approach to this cancer in this work (40.7%) compared to LCC (70.3%), with statistically significant differences (*p* < 0.001). Another result observed is the difference in hospital stay, which is greater in patients operated on for RCC compared to the LCC in a significant way (*p* = 0.04). Despite this, there were no differences in the need for reintervention and readmission between the two groups (*p* = 0.09 and *p* = 0.54, respectively).

In the most novel aspect of this work, the analysis of survival, the results obtained in overall survival, always higher in the RCC group, are comparable to those described in previous studies [22]. As for the breakdown by stages, there are articles published [26] that observe that this difference is independent of the tumor stage or that these differences are only significant in stage III in favor of LCC [14,27]. However, in this study, significant differences were found in favor of LCC in the early stages of the disease (I and II), as is observed in a study carried out in Germany, stating that RCC has a worse prognosis even in stage I of the disease [15].

Based on the care and surgical results, we agree with what experts in colon cancer screening say: improvement of the prognosis can occur mainly by reducing the specific mortality of colon cancer by achieving early detection because they have a better prognosis and because their mortality is reduced by increasing their survival [28]. This strategy is more transcendent in RCC because it is more frequent with the increase in the age of the patients, it is associated in a significant way to more complications (OR 2) and it has more mortality in RCC, as they are larger, more differentiated and aggressive local tumors even in early stages (I and II), as has also described been by other authors [14]. Furthermore, it would also mean a reduction in hospital costs by reducing hospital stays as the main component of the expenditure associated with surgery for this cancer [29].

The main limitation of this study is the size of the sample; however, it is counterbalanced by its main strength, as is the 8-year prospective follow-up period and the 1:1 matching of the sample. This is an aspect to be considered for the period of follow-up of the patients versus previous work.

This study includes a breakdown by causes of mortality, neoplastic or other causes not related to the tumor but rather the presence of conditions, the effectiveness of surgery and care in older patients due to aging. However, taking into account the cause of death, there are no significant differences in the survival of both groups. This increases the soundness of our hypothesis regarding the differences between right and left cancers due to their biological, embryological and genetic potential, and we agree with other professionals that the treatment of this cancer should be stratified by location and age of onset, as well as personalized, which is a current challenge that must be addressed [30].

In addition, prediction models are available to accurately estimate the individual forecast [31].

## 5. Conclusions

First of all, based on the analysis of survival observed in both neoplasms, it is necessary to improve the effectiveness of population-based screening so that curative surgery has greater significance and can reduce mortality rates presented by both entities and improve the quality of life of these patients. Moreover, this is much more important in elderly patients, as these people present more postoperative problems, longer stays and shorter survival, being more relevant in patients with RCC, where there is evidence of higher mortality even in early stages.

Secondly, and in relation to colon cancer care, we must continue to insist on the analysis of the differences between right and left colorectal cancer in order to improve surgical-care results and value the opportunity that minimally invasive approaches represent in both types of cancers, as these results show, to reduce the number of complications and hospital length of stay. All of this will contribute to the improvement of the effectiveness and efficiency of the treatment of colorectal cancer, given its frequency and importance.

## Figures and Tables

**Table 1:** Demographics.

	All (n)	RCC	LCC	*p*
Sex				0.26
Male	232 (58.3%)	110 (55.3%)	122 (61.3%)	
Female	166 (41.7%)	89 (44.7%)	77 (38.7%)	
Age				<0.001
		73.6 (66.9–79.5)	69.6 (61.5–76.72)	
Comorbidities				0.08
Yes		157 (78.9%)	141 (70.8%)	
1	129 (43.3%)	67 (42.7%)	62 (44%)	<0.001
2	78 (26.2%)	40 (25.5%)	38 (27%)	
>2	91 (30.5%)	50 (31.8%)	41 (29.1%)	
No		42 (21.1%)	58 (29.1%)	
Previous surgeries				0.05
Yes	140 (35.2%)	80 (40.2%)	60 (30.1%)	
No	258 (64.8%)	119 (59.8%)	139 (69.8%)	
BMI				0.997
		27.68 (24.90–30.38)	27.91 (25.26–30.272)	
	P POSSUM			
morbidity				0.086
	27 (17.83–40.97)		24 (14.55–36.58)	
	P POSSUM			
mortality				0.027
	1.32 (0.78–2.93)		1 (0.58–2.49)	

**Table 2:** Tumor pathology.

	All (n)	RCC	LCC	*p*
Tumor histology				0.06
Adenocarcinoma	372 (93.5%)	181 (91%)	191 (96%)	
Mucinous carcinoma	23 (5.8%)	15 (7.5%)	8 (4%)	
Others	3 (0.8%)	3 (1.5%)	0	
Lymph nodes isolated				<0.001
<12	59 (14.9%)	18 (9.1%)	41 (20.6%)	
12	338 (85.1%)	180 (90.9%)	158 (79.4%)	
Positive lymph nodes				0.857
Yes	128 (43.1%)	63 (31.8%)	65 (32.7%)	
No	169 (56.9%)	135 (68.2%)	134 (67.3%)	
Stage				0.35
Local (I–IIa)	244 (61.3%)	127 (63.8%)	117 (58.8%)	
Advanced (>IIb)	154 (38.7%)	72 (36.2%)	82 (41.2%)	
Specific Staging				0.71
I	101 (25.4%)	53 (26.6%)	48 (24.1%)	
IIa	142 (35.7%)	74 (37.2%)	68 (32.2%)	
IIb	9 (2.3%)	3 (1.5%)	6 (3%)	
IIc	7 (1.8%)	3 (1.5%)	4 (2%)	
IIIa	15 (3.8%)	6 (3%)	9 (4.5%)	
IIIb	67 (16.8%)	34 (17.1%)	33 (16.5%)	
IIIc	22 (5.5%)	13 (6.5%)	9 (4.5%)	
IVa	33 (8.3%)	12 (6%)	21 (10.6%)	
IVb	2 (0.5%)	1 (0.5%)	1 (0.5%)	

**Table 3:** Postoperative course.

	All (n)	RCC	LCC	*p*
Complications				<0.001
Yes	153 (38.4%)	101 (50.8%)	52 (26.1%)	
No	245 (61.6%)	98 (49.2%)	147 (73.9%)	
CLAVIEN				<0.001
None	246 (61.8%)	98 (49.2%)	148 (74.4%)	
I–II	115 (28.9%)	77 (38.7%)	38 (19.1%)	
>II	37 (9.3%)	24 (12.1%)	13 (6.5%)	
Surgical site infection				<0.001
Yes	44 (11.1%)	32 (16.1%)	12 (6%)	
No	354 (88.9%)	167 (83.9%)	187 (94%)	
Intraabdominal abscess				0.02
Yes	16 (4%)	13 (6.5%)	3 (1.5%)	
No	382 (96%)	186 (93.5%)	196 (98.5%)	
Incisional hernia				0.11
Yes	20 (5%)	14 (7%)	6 (3%)	
No	378 (95%)	185 (93%)	193 (97%)	
Anastomotic leak				0.34
Yes	30 (7.5%)	18 (9.1%)	12 (6%)	
No	368 (92.5%)	181 (91%)	187 (94%)	
Postoperative ileus				0.00
Yes	81 (20.4%)	56 (28.1%)	25 (12.6%)	
No	317 (79.7)	143 (71.9%)	174 (87.4%)	
Reintervention				0.09
Yes	18 (4.5%)	13 (6.5%)	5 (2.5%)	
No	380 (95.5%)	186 (93.5%)	194 (97.5%)	
Readmission (month)				0.54
Yes	11 (2.8%)	7 (3.5%)	4 (2%)	
No	387 (97.2%)	192 (96.5%)	195 (98%)	
Length of stay (days)	8 (10.447)	13.61 (11.95)	9.36 (8.181)	0.004

**Table 4:** Survival analysis.

	Global Survival	N	%	Deaths	N	%
6 months		371	93.2		27	6.8
12 months		340	85.4		58	14.6
	Survival location	6 months	IC 95%	12 months		IC 95%
	RCC	90.5%	85.7–93.6		80.4%	75.5–85.8
	LCC	96.0%	93.4–96.7		90.5%	87.1–94.8
	Global	93%	90.9–95.7		86.2%	82.9–89.5
*Mantel-Cox*				Chi-square		
	RCC/ICC	*p* = 0.005 *		*p* = 0.007 **		

* Differences between CCR and LCC; ** Only in Local Cancer.

## Data Availability

Data are available in an accessible repository that does not issue DOIs. They belong to the hospital registry. They can be consulted with previous consent.
